# Synthesis, Characterization and Reactivity Ratio Study of Poly(di(tri-*n*-butyltin) citraconate-*co*-*N*-vinylimidazole)

**DOI:** 10.3390/molecules15074750

**Published:** 2010-07-07

**Authors:** Salem S. Al-Deyab, Mohamed H. El-Newehy, Ali M. Al-Hazmi

**Affiliations:** Petrochemical Research Chair, Chemistry Department, College of Science, King Saud University, Riyadh 11451, P.O. Box 2455, Saudi Arabia

**Keywords:** bis(tri-*n*-butyltin) oxide, citraconate, *N*-vinyl imidazole, reactivity ratio

## Abstract

The organotin monomer di(tri-*n*-butyltin) citraconate (DTBTC, **I**) was synthesized. Subsequently this monomer was copolymerized with *N*-vinylimidazole (VI) using a free radical technique. The overall conversion was kept low (≤14% wt/wt) for all studied samples and the copolymer composition was determined from tin analysis using the Gilman and Rosenberg method. The synthesized monomer and copolymer were further characterized by elemental analysis, ^1^H- and ^13^C-NMR, and FTIR spectroscopy.

## 1. Introduction

A copolymer’s composition is an important factor in the evaluation of its utility [1,2,3,4]. Controlling the polymer property parameters, such as copolymer composition and sequence distribution and molecular weight averages, is of particular importance in copolymerization processes [2]. In order to calculate the rate of polymerization or polymer productivity and copolymer composition, monomer reactivity ratios must be known [5]. Reactivity ratios are among the most important parameters for the composition equation of copolymers, as they can offer information such as the relative reactivity of monomer pairs and help estimate the copolymer composition [2,3]. Determination of the monomer reactivity ratios with small confidence intervals requires sensitive analytical techniques, careful planning of experiments and the use of statistically valid methods of estimation [5,6]. The method which is used most often nowadays for estimating monomer reactivity ratios is to perform low conversion copolymerization at various initial monomer feed compositions. Subsequently, the copolymer composition is determined for each reaction. Traditional methods for estimating monomer reactivity ratios are based on, first, transforming the instantaneous copolymer composition equation into a form that is linear in the parameters *r*_1_ and *r*_2_ and then estimating the monomer reactivity ratios by graphical plotting or by the linear least-squares method [7,8,9,10]. Linearization of the copolymer composition equation will distort the error distributions associated with the data.

In this paper di(tri-*n*-butyltin) citraconate (DTBTC, **I**) was synthesized for use as an organotin monomer. This monomer was then copolymerized with *N*-vinylimidazole (VI). The structural characterization of the copolymer was performed and the reactivity ratios in the copolymerization determined for the classical copolymerization model using the Finemann–Ross linearization method (FR method) [1,2,11].

## 2. Results and Discussion

### 2.1. Monomer Synthesis

**Scheme 1:**
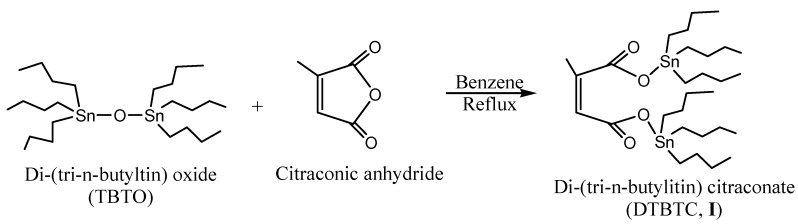
Synthesis of di-(tri-n-butyltin)citraconate(DTBTC, **I**).

Di(tri-*n*-butyltin) citraconate (DTBTC, **I**) was prepared using equimolar ratios of bis(tri-*n*-butyltin) oxide (TBTO) and citraconic anhydride (Scheme 1). The purity of the prepared monomer was checked by Thin Layer Chromatography (TLC) using chloroform as eluant. In addition, the structure was confirmed by elemental analysis, FTIR, ^1^H- and ^13^C-NMR spectroscopy.

The FTIR spectrum of DTBTC **(I) **showed characteristic peaks at 2,852, 2,868, 2,922, and 2,954 cm^-1^ assigned to C-H stretching (-CH_2_CH_2_CH_2_CH_3_, & -CH=CCH_3_-), at 1,750 and 1,700 cm^-1^ assigned to C=O stretching and at 1,642 cm^-1^, assigned to C=C stretching. Moreover, the disappearance of anhydride group stretching peak at 1,850 cm^-1^ confirmed the complete conversion of the anhydride group into ester groups.

The ^1^H-NMR Spectrum (CDCl_3_) of DTBTC **(I) **showed peaks at δ 0.86 (triplet, -CH_2_CH_2_CH_2_C*H_3_*), 1.26-1.30 (multiplet, -CH_2_C*H_2_*CH_2_CH_3_), δ 1.34-1.61 (multiplet, -C*H_2_*CH_2_C*H_2_*CH_3_), δ 1.96 (singlet, ‑CH=CC*H_3_*-), 5.73 (singlet, -C*H*=CCH_3_-), while the corresponding ^13^C-NMR spectrum (CDCl_3_) showed peaks at δ 13.69, 16.55, 27.81, 28.00 (-*C*H_2_*C*H_2_*C*H_2_*C*H_3_), 20.00 (-CH=C*C*H_3_-), 120.51 and 145.31 (-*C*H=*C*H-), 170.50 (-CH=CCH_3_-*C*=O) and 174.62 (O=*C*-CH=CCH_3_-). The calculated (measured) elemental microanalyses results were in a good agreement [%C: 49.18 (49.73); %H: 8.25 (8.95): %Sn: 33.25 (33.10)]. Tin was estimated using the Gilman and Rosenberg method [12].

### 2.2. Copolymerization Method

**Scheme 2:**
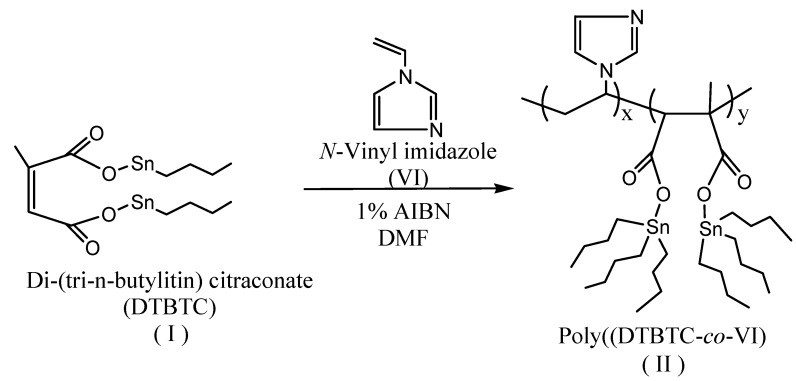
Synthesis of Poly(di(tri-*n*-butyltin) citraconate-*co-N*-vinylimidazole).

Poly(DTBTC-*co*-VI) (**II**) was prepared in DMF solution with a total concentration of 6.67 mol/*via* a free radical technique using AIBN as initiator at 60 ºC L for different time intervals (Scheme 2). The copolymerization was stopped at overall conversions ≤14% wt/wt and the formed copolymer (**II)** was precipitated in an excess amount of acetone, and then dried in an oven under vacuum at 60 ºC.

Copolymer (**II)** was characterized by FTIR and ^1^H-NMR spectroscopy. After 3 h the FT-IR spectrum of (**II)** with an overall conversion of 7.81%, was characterized by the disappearance of the C=C stretching bands at 1,650 and 1,670 cm^-1^ of DTBTC and VI, respectively, which confirm the formation of the copolymer. The FTIR spectrum showed peaks at 1,592, 1,627 and 1,657 cm^-1^ assigned to C=C stretching of the imidazole ring and characteristic peaks at 1,605 and 1,766 cm^-1^ assigned to C=O stretching. In addition the spectrum showed peaks at 2,850, 2,924, 3,025, 3,058, and 3,080 cm^-1^ assigned to aliphatic C-H stretching. The ^1^H-NMR spectrum (CDCl_3_) of (**II)** was characterized by the disappearance of the peaks at δ 5.49–6.04 ppm (-C*H*=C-, -C*H*=CCH_3_- and C*H_2_*=C*H*-) corresponding to DTBTC and VI, respectively, which further confirm the formation of the copolymer. The spectrum of (**II)** was characterized by the presence of peaks at δ 0.85-1.43 ppm (-C*H_2_*C*H_2_*C*H_2_*C*H_3_*), 1.83 ppm (-C*H_2_*-CHN-), 2.87 ppm -C-C*H*COOSn-), 4.53 ppm (-CH_2_-C*H*N-), and at 6.90-7.35 ppm (*H_arom_*, imidazole ring).

### 2.3. Reactivity Ratio Determination

**Figure 1:**
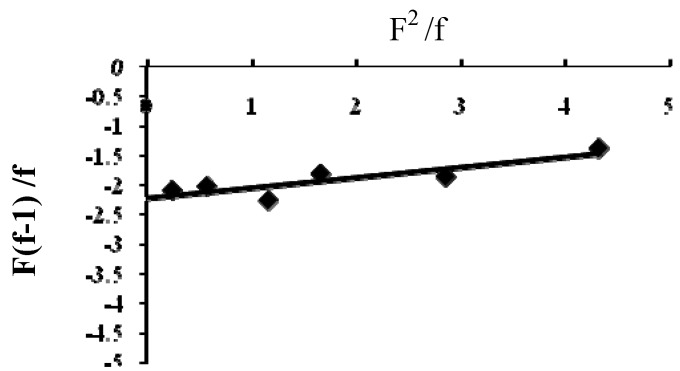
F(f-1)/f vs F^2^/f plot for Poly(DTBTC-*co*-VI) (**II**).

Different copolymers with different ratios were prepared and the percentage of nitrogen was measured, and subsequently the copolymer composition (f) was determined, as shown in Table 1. The monomer reactivity ratios and the content of the reaction mixture and the copolymer were calculated according to the FR method [8,9,10] (Table 2). The FR parameters for DTBTC and VI were calculated by plotting the relation between F(f-1)/f and F^2^/f, as shown in Figure 1.

**Table 1:** The experimental percentage of nitrogen of poly(DTBTC-*co*-VI) (**II**) with different ratios and its composition parameters.

Copolymer Ratio	%N	M_1_^a^	F^b^	m_1_^c^	f^d^	Conversion (% wt/wt)^f^
10/90	21.60	0.1	0.11	0.048	0.05	9.63
20/80	16.34	0.2	0.25	0.099	0.11	6.91
30/70	13.29	0.3	0.43	0.14	0.16	9.98
40/60	9.75	0.4	0.67	0.21	0.27	11.5
50/50	8.18	0.5	1.0	0.25	0.35	13

^a^ Mole fraction of DTBTC in reaction mixture; ^b ^Molar ratio of DTBTC to **VI** in reaction mixture; ^c ^Mole fraction of DTBTC in copolymer; ^d ^Molar ratio of DTBTC to **VI** in copolymer; ^f ^Overall conversion.

**Table 2:** The monomer reactivity ratios and the FR parameters of poly(DTBTC-*co*-VI) (**II**).

Copolymer Ratio	Monomer Ratio F = M_1_/M_2_	M-Unit Ratio in Copolymer	Parameters of the FR Eq.	
			F^2^/f	F/f(f-1)
10/90	0.11	0.05	0.24	-2.09
20/80	0.25	0.11	0.57	-2.02
30/70	0.43	0.16	1.16	-2.26
40/60	0.67	0.27	1.66	-181
50/50	1.0	0.35	2.86	-1.86

As r_1_r_2_<1 and r_1_r_2_ are close to zero, this implies that monomers tend to form alternating copolymers [13]. From the fact that the values of the experimental reactivity ratio, r_1_ (*k_11_/k_12_*) are smaller than r_2_ (*k_22_/k_21_*), it is evident that the DTBTC monomer (r_1 _= 0.1697) is less reactive towards the addition of its units compared to the addition of VI units, while VI (r_2 _= 2.2094) is more reactive towards the addition of its units, compared to the addition of DTBTC units.

## 3. Experimental

### 3.1. Materials

Citraconic anhydride and bis(tri-*n*-butyltin) oxide were purchased from Fluka. Benzoyl peroxide was purchased from BDH. 2,2^'^-Azobisisobutyronitrile (AIBN) was purchased from Riedel-De Haën. All solvents were purchased from BDH and used as received.

### 3.2. Characterization

^1^H- and ^13^C-NMR Spectra were recorded on a JEOL (400 MHz) instrument. FTIR spectra were recorded on a Perkin Elmer 883 spectrophotometer. Elemental analyses were performed on a Perkin Elmer Series II CHN/O Analyzer 2400. Thin-layer chromatography (TLC) was performed using the ascending technique with silica gel 60F 254 precoated aluminum sheets.

### 3.3. Synthesis of Di(tri-n-butyltin) citraconate (DTBTC, ***I***)

The title monomer di(tri-*n*-butyltin) citraconate (DTBTC, **I**) was prepared according to Al-Diab *et al.* [1]. The method can be summarized as follows: bis(tri-*n*-butyltin oxide (14.9 g, 25.0 mmol) was added to citraconic anhydride (2.8 g, 25.0 mmol), and benzene (150 mL) in a 500 mL round bottom flask. The reaction mixture was refluxed with stirring for 4 h. The solvent was evaporated on a rotavapor to give pale yellow viscous oil (17.0 g, 95.38% yield).

### 3.4. Copolymerization Method

In three-necked round bottomed flask, AIBN (1.5 × 10^-2^ mol/L) was dissolved in DMF (25 mL), and then the calculated molar quantities of the monomers were added to a concentration of 2.5 mol/L. The copolymerization mixture was spurge with nitrogen to expel oxygen and then the copolymerization was done at 60 ºC for the desired period of time. The copolymer formed was precipitated in acetone and was dried in an oven under vacuum at 60 ºC. For reactivity ratio determination, the copolymerization was stopped at overall conversions below 14% wt/wt [14] calculated from the total weight of both monomers by changing the time of polymerization [15].

### 3.5. Reactivity Ratios Determination

For reactivity ratio determination, copolymerization was performed with different initial feed ratios while maintaining the monomer conversion below 10%. The Fineman–Ross (FR) method was employed. The initiator concentration was kept at 1% relative to the total monomers concentration in benzene or DMF. Monomer reactivity ratios can be calculated from the experimental results depending on the copolymer composition. Copolymer composition can be expressed as follows:

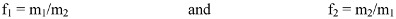

where m_1_ and m_2_ are the mole fractions of DTBTC and vinyl imidazole in the copolymer, respectively, and f_1_ and f_2_ are its molar ratios in the copolymer.

Moreover, the feed composition of the reaction mixture is known in advance, so feed composition was used in the calculation of the reactivity ratios and can be expressed as follows:

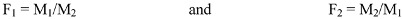

where M_1_ and M_2_ are the mole fractions of DTBTC and vinyl imidazole in the reaction mixture, respectively, and F_1_ and F_2_ are its molar ratios in the feed composition.

In this research, the calculations were based on the tin content in the copolymer composition [16,17]. The Fineman–Ross (FR) [1,2,11] method is based on the use of copolymer composition and the content of the polymerization mixture. Based on the calculations of the copolymer composition and feed composition and according to the following equation:

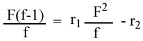

a plot of (F^2^/f) on X-axis vs {F(f-1)/f} on Y-axis gave a straight line, whose intercept is r_2_ and the slope is r_1_.

## 4. Conclusions

The organotin monomer, di(tri-*n*-butyltin) citraconate (DTBTC, **I**) was synthesized. This monomer was copolymerized with *N*-vinylimidazole (VI) *via* a free radical technique. The overall conversion was kept low (≤14% wt/wt) for all studied samples and the copolymer composition was determined from nitrogen analysis. As r_1_r_2_<1, the copolymer tends to form alternating copolymers as r_1_r_2_ is close to zero. From the values of the experimental reactivity ratio, r_1_ is smaller than r_2_, indicating that the DTBTC monomer is less reactive towards the addition of its units compared to the addition of VI units. On the other hand, compound VI is more reactive towards the addition of its units compared to the addition of DTBTC units.
